# Material Aspects of Thin-Film Composite Membranes for CO_2_/N_2_ Separation: Metal–Organic Frameworks vs. Graphene Oxides vs. Ionic Liquids

**DOI:** 10.3390/polym16212998

**Published:** 2024-10-25

**Authors:** Na Yeong Oh, So Youn Lee, Jiwon Lee, Hyo Jun Min, Seyed Saeid Hosseini, Rajkumar Patel, Jong Hak Kim

**Affiliations:** 1 Department of Chemical and Biomolecular Engineering, Yonsei University, 50 Yonsei-ro, Seodaemun-gu, Seoul 03722, Republic of Korea; ony2499@yonsei.ac.kr (N.Y.O.); syl9336@gmail.com (S.Y.L.);; 2 Energy and Environmental Science and Engineering (EESE), Integrated Science and Engineering Division (ISED), Underwood International College, Yonsei University, 85 Songdogwahak-ro, Yeonsu-gu, Incheon 21983, Republic of Korea; 3 Department of Chemical Engineering, Vrije Universiteit Brussel, Pleinlaan 2, 1050 Brussels, Belgium; seyedsaeidhosseini@gmail.com

**Keywords:** thin-film composite (TFC), CO_2_ separation membrane, metal–organic frameworks (MOFs), graphene oxides (GOs), ionic liquids (ILs)

## Abstract

Thin-film composite (TFC) membranes containing various fillers and additives present an effective alternative to conventional dense polymer membranes, which often suffer from low permeance (flux) and the permeability–selectivity tradeoff. Alongside the development and utilization of numerous new polymers over the past few decades, diverse additives such as metal–organic frameworks (MOFs), graphene oxides (GOs), and ionic liquids (ILs) have been integrated into the polymer matrix to enhance performance. However, achieving desirable interfacial compatibility between these additives and the host polymer matrix, particularly in TFC structures, remains a significant challenge. This review discusses recent advancements in TFC membranes for CO_2_/N_2_ separation, focusing on material structure, polymer–additive interaction, interface and separation properties. Specifically, we examine membranes operating under dry conditions to clearly assess the impact of additives on membrane properties and performance. Additionally, we provide a perspective on future research directions for designing high-performance membrane materials.

## 1. Introduction

Reducing greenhouse gas emissions has become a key objective of the current era. CO_2_ capture and storage (CCS) is one of the primary methods employed to reduce CO_2_ emissions from power plants [1,2,3,4,5]. Among the various CCS techniques, chemical absorption using an amine-based sorbent is the most widely adopted [6,7,8,9,10,11,12]. However, this method requires substantial space and high energy for operation. Its implementation is further constrained by operational complexities, including a high risk of corrosion and safety concerns. In contrast, membrane separation offers benefits such as lower energy consumption and milder operational conditions [13,14,15,16,17,18]. The performance of membranes is largely determined by two critical factors: permeability and selectivity. Unfortunately, a trade-off between these two factors is observed in most conventional polymeric membranes, known as the Robeson upper bound limit [19,20,21,22,23,24,25]. Consequently, extensive efforts have been made to enhance both permeability and selectivity simultaneously by incorporating additives such as a metal–organic framework (MOF) [26,27,28,29,30,31,32,33,34,35], graphene oxide (GO) [36,37,38,39,40,41,42,43], and ionic liquid (IL) [44,45,46,47,48,49,50,51,52] into the polymer matrix. It is a common practice to combine two or more types of additives to enhance the separation performance of membranes [53,54,55,56,57,58,59,60,61,62,63].

For gas separation membranes, permeability is expressed in barrers (1 barrer = 1 × 10^−10^ cm^3^ (STP) cm cm^−2^ s^−1^ cmHg^−1^), while permeance is given in GPUs (1 GPU = 1 × 10^−6^ cm^3^ (STP) cm^−2^ s^−1^ cmHg^−1^). Both parameters are crucial for membrane applications. Gas permeability indicates the intrinsic property of the material, as it is normalized by the membrane thickness [64,65,66,67,68]. In contrast, gas permeance reflects the membrane’s overall performance and is directly related to membrane flux. In order to achieve high GPU values, the thickness of the selective layer must be reduced without affecting the selectivity nor resulting in defective spots. Thin-film composite (TFC) membranes are the most efficient type of membranes having a selective layer of low thickness (typically less than 1 μm) [69,70,71,72,73,74,75]. The TFC membrane comprises a thin, dense, and selective layer coated on a high-permeance (typically more than 1 × 10^4^ GPU), porous support layer. The selective layer can effectively separate CO_2_ from other gases (e.g., N_2_ or CH_4_) owing to the higher diffusivity and solubility of CO_2_ molecules.

Over the past few decades, TFC-containing additives or porous fillers have received considerable attention because of their high permeance (GPU) and high processability, but they frequently lead to the formation of interfacial defects (or voids) between the filler and polymer matrix; this issue aggravates with decreasing membrane thickness [76,77,78,79,80,81,82,83]. Additives were included in the dense polymer selective layer to improve the separation performance of the membrane. A recent study offers an overview of various emerging nanomaterials that have shown promising results for gas separation and pervaporation applications [84]. These nanomaterials provide significant benefits, such as high stability, high porosity, and tunable surface properties and structures [85,86,87,88,89,90].

**Figure 1:**
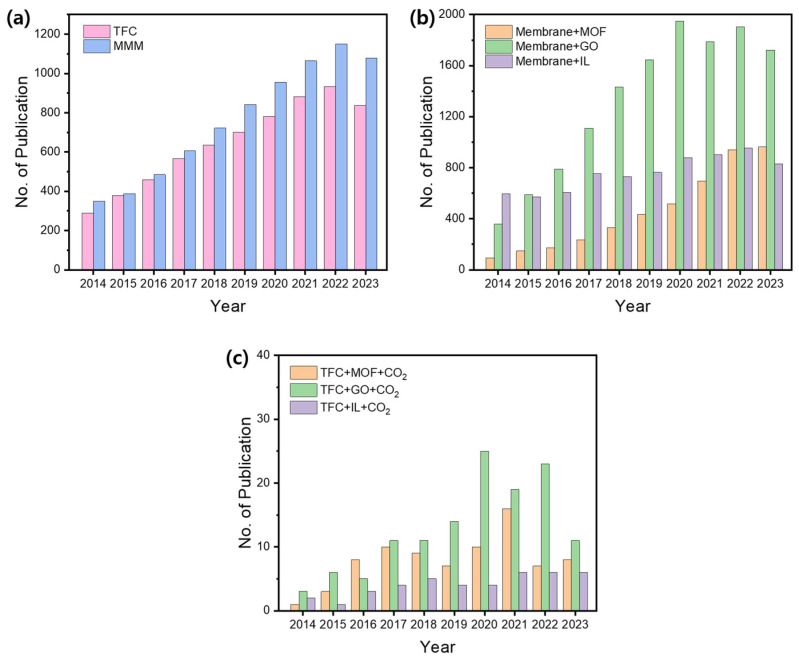
Number of published papers on (**a**) TFC membrane and MMM, (**b**) membranes with three types of additives (MOFs, GOs, and ILs), and (**c**) TFC membranes with three types of additives (MOFs, GOs, and ILs) for CO_2_ separation over the last ten years (2014–2023), with data obtained from the Web of Science (https://www.webofscience.com/wos/woscc/basic-search, accessed on 9 September 2024).

**Figure 2:**
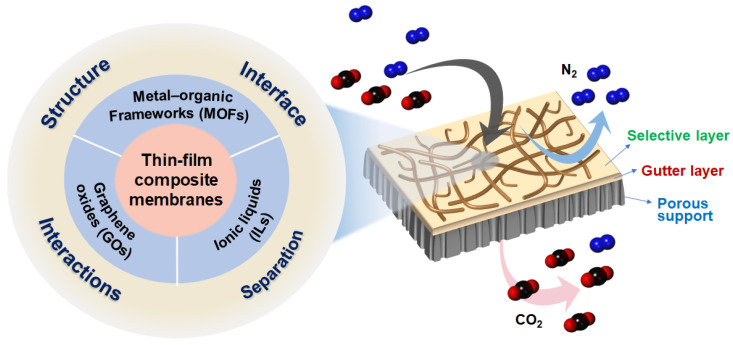
A schematic diagram illustrating the focus of this review paper on TFC membranes incorporating three types of additives for CO_2_/N_2_ separation. Typical TFC membranes for CO_2_ separation are composed of a top selective layer, a middle gutter layer, and a bottom porous layer.

The first viable TFC membrane was fabricated for reverse osmosis applications in 1972 by John Cadotte at North Star Technologies (later FilmTec Corporation) using interfacial polymerization of polyamide on a porous polymeric support. Since then, TFC membranes have received much attention, mainly in the field of desalination, with the number of papers on this topic reaching nearly 300 in 2014 (Figure 1a). Mixed-matrix membrane (MMM) has also received great attention in various fields, including the gas separation field, and the number of papers on MMM has continued to increase from 350 in 2014 to more than 1100 in 2022. A literature survey indicates that there were approximately 1000 papers in 2014 focused on membranes incorporating at least one of the three types of additives: MOFs, GOs, and ILs. Since then, the number of publications in this area has grown significantly (Figure 1b). Notably, research on membranes with MOF and GO has grown rapidly, with GO-based membranes seeing the most significant increase. As shown in Figure 1c, fewer papers have been published on TFC membranes compared with free-standing dense membranes for CO_2_ separation. This is attributed to the great challenge of producing defect-free, high-performance TFC membranes that offer both high gas permeance and selectivity. Despite this challenge, the number of publications on TFCs has steadily increased in subsequent years. Although there were some review papers on MMM or TFC membranes for CO_2_ separation [5,64,66,67,71,85], there was no systematic review on TFC-MMMs under non-humidified conditions. Humidified wet membranes utilizing facilitated transport have been extensively studied because water vapor in the feed gas significantly boosts separation performance by enhancing both permeability and selectivity [91,92,93,94]. However, failure to carefully control humidity and temperature often results in water condensation, which can negatively affect separation performance and long-term stability. Therefore, addressing the practical challenges of high sensitivity to humidity and temperature fluctuations is essential for real-world applications. In this review, we focus on recent advancements in the fabrication of dry TFC membranes for CO₂/N₂ separation under non-humidified conditions, aiming to clearly assess the impact of additives on membrane properties and performance. We also highlight the challenges addressed so far and the improvements achieved in separation performance. Our emphasis was on three types of additives (MOFs, GOs, and ILs), as they represented the most commonly utilized ones for TFC membranes designed for CO_2_ separation (Figure 2).

## 2. MOF-Containing TFC Membranes

MOF materials have a high gas separation potential and have been widely utilized owing to their large specific surface area, facile modification ability, molecular sieving effect, tunable pore size, and controllable chemical affinity through functional group selection [95,96,97,98,99,100,101,102,103,104,105]. In particular, the zeolite imidazole framework (ZIF-8), a widely utilized MOF, is well dispersed in mild solvents such as alcohol and can be easily synthesized [106]. The addition of ZIF-8 provided the membrane with a more permeable selective layer and higher thermal durability. Introducing an MOF layer into a TFC membrane requires stable adhesion to the flexible support layer and the formation of a uniform and cohesive film [107]. MMMs incorporating flexible MOFs face the fundamental question of how the intrinsic expansion of the flexible MOF affects the structural phase transition, adsorption amounts, and permeation properties [108]. The key criteria for selecting MOFs include high CO₂ adsorption capacity, suitable pore size, optimal particle size, good dispersion in solvent, ease of functionalization, low cost and scalability, and strong compatibility with the polymer matrix.

**Figure 3:**
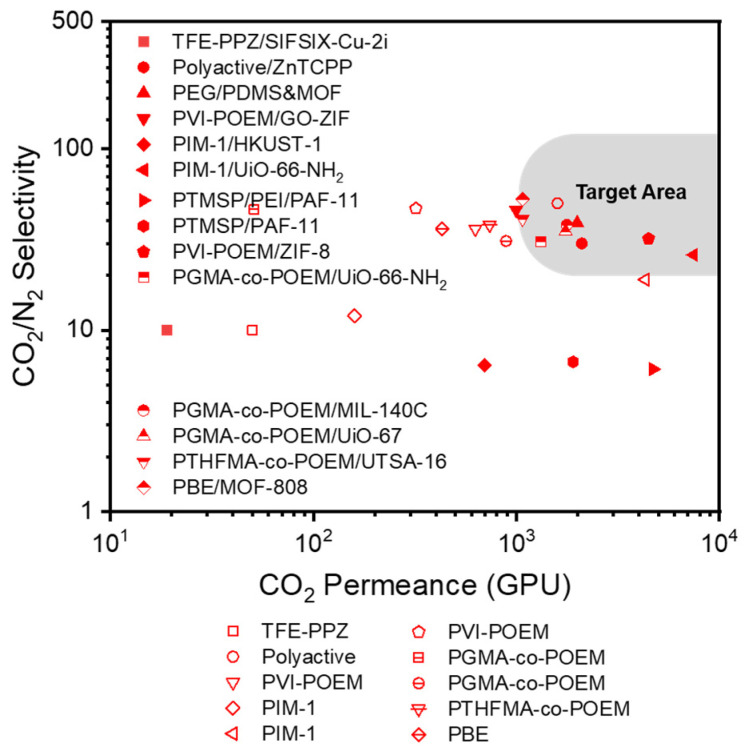
CO_2_/N_2_ selectivity vs. CO_2_ permeance of dry TFC-MMMs with various MOF fillers reported in the literature. Unfilled symbols represent TFC membranes without the addition of MOF fillers.

**Figure 4:**
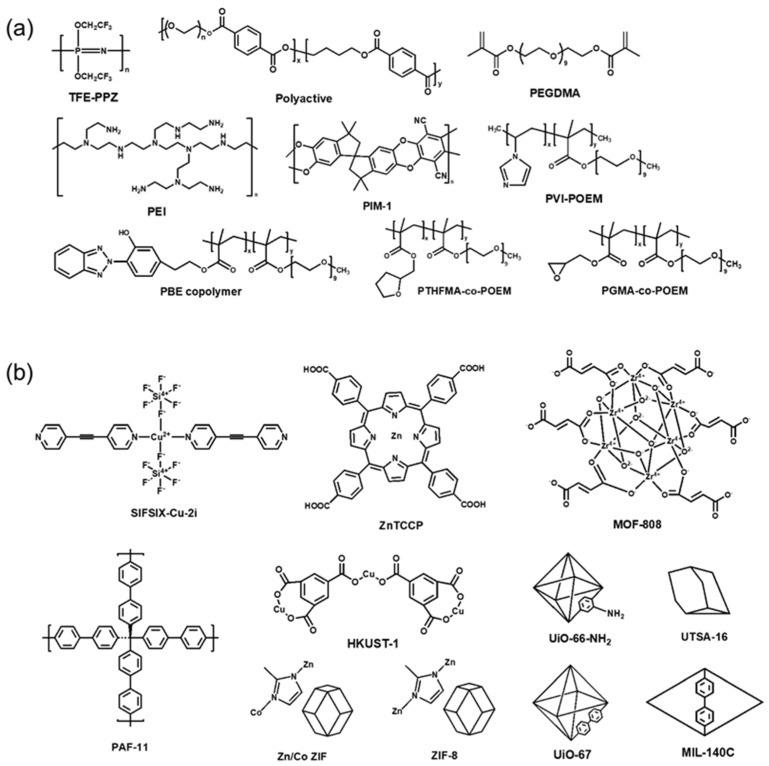
Chemical structures of (**a**) polymer and (**b**) various MOFs used in the selective layer shown in Table 1 and Figure 3.

The CO_2_/N_2_ separation performance of dry TFC-MMMs with various MOF fillers reported in the literature are shown in Figure 3 and Table 1. The chemical structures of polymers and MOFs are shown in Figure 4. Some MMMs containing MOFs showed excellent separation performance, which lies in the commercial target area for CO_2_/N_2_ separation. The majority of research papers have employed a gutter layer, such as PTSMP or PDMS, positioned between the top selective layer and the bottom porous support. This intermediate gutter layer prevents the deep penetration of the selective layer into the porous layer. Without this gutter layer, such penetration would cause a significant reduction in gas permeability or selectivity due to the formation of a defective and non-uniform selective layer. Although some studies have demonstrated improved CO_2_ solubility through the incorporation of MOFs, the primary reason for the enhanced performance is the increased diffusivity through the MOF pores. Membrane thickness ranges from 50 nm to 500 nm, typically remaining under 1 μm. Reducing thickness is desirable for achieving higher gas permeance (in GPU), but it poses a challenge to maintain selectivity, especially in TFC membranes. This is because achieving close contact between the porous MOF filler and the polymer matrix becomes difficult at submicron thicknesses, highlighting the critical importance of improved interfacial contact in TFC membranes.

The first automated flue gas permeation membrane testing system for post-combustion was reported by the National Carbon Capture Center, supported by the Department of Energy, USA [109]. In this study, large-scale membrane skids were fabricated using dense films of PDMS and poly(bistrifluoroethoxyphosphazene) (TFE-PPZ) coated on Torlon^®^ fibers. Specifically, MOF particles, [Cu(4,4′-dipyridylacetylene)_2_(SiF_6_)]_n_, known as SIFSIX-Cu, were incorporated into the TFE-PPZ matrix to form hollow fiber MMMs. Despite the excellent materials selection of polymer and MOF, the overall performance was low (CO_2_ permeance: 19–55 GPU, CO_2_/N_2_ selectivity < 15), highlighting the significant challenges in the commercial application of large-scale, hollow fiber membrane skids for real flue gas mixtures compared with small-scale flat membranes.

In general, MOFs are added to the selective polymer layer to form MMMs, but they can also be used directly as the gutter layer. In one study [110], zinc(II) tetrakis(4-carboxyphenyl)porphyrin) (ZnTCPP) MOF nanosheets, with a thickness of 3–4 nm prepared by the surfactant-assisted method, were used as the gutter layer between Polyactive selective layer and porous polyacrylonitrile (PAN) support. The TFC membrane achieved a high CO_2_ permeance of 2100 GPU and a moderate CO_2_/N_2_ selectivity of 30. The ultrathin (~25 nm) flat MOF layer showed reduced gas resistance compared with conventional PDMS. However, there was no direct comparison with the widely used PTMSP gutter layer. The membranes exhibited excellent mechanical strength, capable of operating at elevated pressures (6.0 bar) over prolonged periods, but there was no mention of the possibility of large-area membrane fabrication.

The PDMS gutter layer often encounters resistance issues related to thickness-dependent gas permeability and geometric restriction due to the confined pore accessibility of the porous support. In a study [111], MOFs were incorporated into a PDMS gutter to address these problems. The use of MOF was effective in increasing the free volume, mitigating pore infiltration, and enhancing gas transport speed. The TFC membrane, with a poly(ethylene glycol) (PEG) selective layer prepared via surface-initiated atom transfer radical polymerization onto the PDMS/MOF gutter layer, exhibited excellent separation performance, achieving a CO_2_ permeance of 1990 GPU and CO_2_/N_2_ selectivity of 39.

Another study investigated the effects of incorporating Mg-MOF-74, aluminum terephthalate (MIL-53), hexafluorotitanate (TIFSIX-3), and Zn_2_(bim)_4_ into a PIM-1 polymer [120]. These MOF structures had pore sizes of 0.2–1.1 nm, suitable for size sieving small gas molecules. Among them, Mg-MOF-74 could adsorb more CO_2_ than other gases. The MMM consisting of PIM-1/MOF-74 showed improved physical aging properties due to the exfoliation of the layered particles in the PIM-1 layer and the formation of an extensive polymer/particle interface.

In another study, a TFC-MMM was fabricated with ZIF-decorated GO as a filler dispersed in an amorphous copolymer matrix, poly(vinyl imidazole)-*co*-poly(oxyethylene methacrylate) (PVI-POEM) [53]. The membrane featured a PTMSP gutter layer and a PSf support layer. The GO-ZIF filler, synthesized by bonding metal ions with 2-methylimidazole (2MI), had an appropriate pore size for separating small CO_2_ molecules from larger ones. The membrane achieved a CO_2_ permeance of 995.1 GPU, a CO_2_/CH_4_ selectivity of 17.5, and a CO_2_/N_2_ selectivity of 45.9. The chemical structure of the organic linker in the ZIF is similar to that of poly(vinyl imidazole), exhibiting comparable polarity and molecular interactions. This study underscores the significance of the molecular structure of PVI-POEM and its adhesive surface, which contributes to exceptional interfacial compatibility. It emphasizes the significance of interfacial engineering between the polymer matrix and fillers in reducing defects in the MMM.

It is interesting to note that a three-dimensional (3D) printing technique was employed in fabricating membranes. In a study [112], PIM-1 was used as the selective matrix and HKUST-1 (also known as MOF-199) as the filler. The effects of casting concentration and the number of electrospraying cycles on membrane thickness and gas separation performance were evaluated. Despite the innovative method for creating TFC-MMMs, the separation performance was rather poor. With a membrane thickness of 2.75 μm, the CO_2_ permeance reached 696 GPU, and the CO_2_/N_2_ selectivity was only 6.4, indicating that this type of membrane is unlikely to be applied in practice.

In a study, an MMM containing MOFs was used as both the selective layer and the gutter layer [113]. The gutter layer consisted of amorphous MOF nanosheets in a PDMS matrix, while the selective layer was prepared using MOF-74-Ni and UiO-66-NH_2_ nanofillers dispersed in a PIM-1 matrix. The resulting TFC-MMMs improved CO_2_ permeance to 4660–7460 GPU and CO_2_/N_2_ selectivity to 26–33 compared with pristine PIM-1. Additionally, the TFC membrane exhibited high resistance to aging, remaining stable even after 8 weeks, demonstrating the multi-functionality and diverse roles of MOFs.

The use of porous aromatic frameworks (PAFs) and covalent organic frameworks (COFs) has recently garnered significant interest in MMMs for gas separation. These materials are emerging as viable alternatives to conventional MOFs due to their excellent molecular sieving properties. A review of the literature reveals that only a few recent studies have explored TFC membranes incorporating these porous materials, suggesting substantial potential for growth in this research area. In one study [114], 10 wt% PAF-11 was integrated into a polydimethylsiloxane (PDMS) thin film on a porous polyacrylonitrile (PAN) support. This membrane demonstrated impressive separation performance for the initial 1000–2000 h, which gradually declined over time. Notably, when the membrane included PAF-11 filler and polyethyleneimine (PEI) crosslinking, it maintained high permeability during prolonged use. Another study investigated aging prevention in a TFC membrane composed of a PTMSP layer with PAF-11 filler [115]. The primary aim of incorporating PAF fillers is to mitigate the physical aging of membranes by restricting macro-chain mobility or increasing the fractional free volume of PTMSP [121]. There are two papers on TFC membranes containing COFs [122,123], but in both cases, the separation performance was evaluated under humid conditions. Therefore, it is unclear whether the improved performance results solely from the COF filler. Despite many advantages, the reported separation performance of TFC membranes containing PAFs or COFs still lags behind that of membranes with MOFs.

In brief, the utilization of TFC-MMMs with various MOF fillers for CO_2_/N_2_ separation is characterized by several key features. Firstly, both hydrophobic PSf and hydrophilic PAN have been widely used as porous supports, indicating that surface hydrophilicity is not significantly important for the support. Instead, surface pore size, overall pore connectivity, and gas permeance are more critical for the support. Secondly, both glassy polymers (e.g., PIM) and rubbery polymers (e.g., PEO-based polymers) served as selective layers. The separation mechanisms differ: glassy polymers rely on diffusivity (kinetic diameter), while rubbery polymers rely on solubility. Thirdly, the gutter layer was primarily made from high-permeance polymers such as PTMSP or PDMS, with only a few studies utilizing MOF layers as the gutter layer. The large-scale application of MOF layers as the gutter layer appears challenging. The gutter layer is used between the top selective layer and the bottom porous support to prevent the selective layer from infiltrating the porous support, which serves to prevent reduction in gas permeance. MOF content varied notably across studies, ranging from 5% to 70%. Generally, separation performance improved with higher filler content due to enhanced diffusivity, although beyond a certain concentration, permeance increased while selectivity decreased due to interfacial voids formed by filler aggregation. Although ZIF, particularly ZIF-8, has the ideal pore aperture (3.4 Å) for separating small CO_2_ (3.3 Å) from large N_2_ (3.64 Å), other MOFs with larger pore sizes have also been extensively utilized and demonstrated excellent separation performance. This suggests that the pore aperture of MOFs can be tuned to be effective in gas separation depending on the polymer matrix type and influenced by the infiltration of polymer chains into the pores.

## 3. GO-Containing TFC Membranes

**Figure 5:**
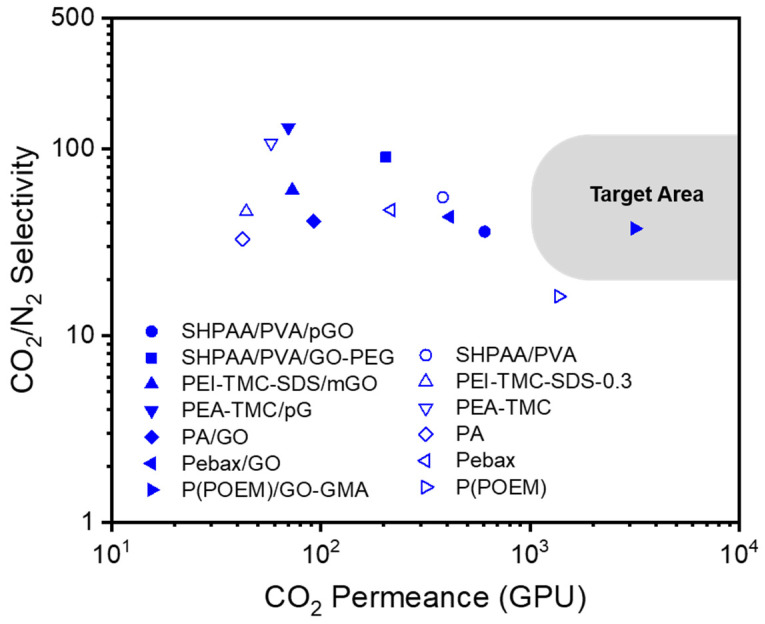
CO_2_/N_2_ selectivity vs. CO_2_ permeance of dry TFC-MMMs with various GOs reported in the literature. Unfilled symbols represent TFC membranes without the addition of GO fillers.

**Figure 6:**
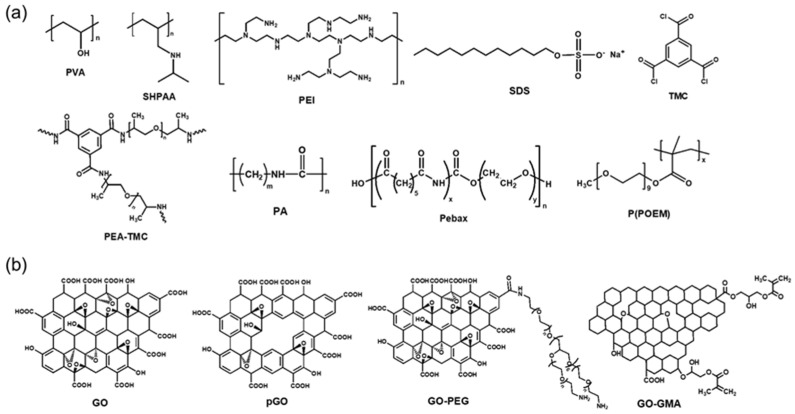
(**a**) Chemical structures of (**a**) polymer and (**b**) various GOs used in the selective layer shown in Table 2 and Figure 5.

Various graphene derivatives have also been incorporated as fillers in polymer matrices to form MMMs. GO with a thickness in the nanometer range and high mechanical strength allows the tuning of intrinsic nanochannels between the adjacent layers of TFC membranes [124,125,126,127,128,129,130,131,132,133]. This allows the effective separation of small gas molecules. In addition, the abundant functional groups of GO can strengthen the interactions between permeants and the membrane. However, some aspects of the GO transport mechanism remain unclear; therefore, further research is required [134,135]. Other derivatives, such as reduced GO (rGO) and graphene quantum dots (GQDs), have also been introduced as fillers. The CO_2_/N_2_ separation performance of dry TFC-MMMs with various GO fillers reported in the literature are shown in Figure 5 and Table 2. The chemical structures of polymers and GOs are shown in Figure 6. Only one MMM containing GOs showed excellent separation performance, which lies in the commercial target area for CO_2_/N_2_ separation.

In one study, a TFC membrane comprising an SHPAA/PVA polymer blend matrix and three types of GO nanofillers was fabricated [136]. Interestingly, both GO and pGO disrupted polymer chain packing and reoriented water distribution, leading to increased CO_2_ sorption even at a very low loading of 0.2 wt%. In contrast, when GOs were modified with PEG chains (GO-PEG), the diffusional resistance increased, resulting in lower CO_2_ permeance due to the rigidified amino groups. Therefore, there is a tradeoff between permeance and selectivity: the pGO-based membrane exhibited high permeance and low selectivity, while the GO-PEG-based membrane showed the opposite.

Similar to the method employed for reverse osmosis, TFC-MMMs can be fabricated by interfacial polymerization for CO_2_ separation. In one study [137], PEG and PDA were mixed and subjected to a spinning process to improve the affinity between the support and coating solution in a hollow fiber membrane. PEI served as an aqueous monomer, trimesoyl chloride (TMC) as an organic monomer, and sodium dodecyl sulfate (SDS) as a surfactant for treating the support surface. In particular, SDS plays a pivotal role in enhancing the reactivity of the reactants, thereby improving the permeance and selectivity for CO_2_ transport. The CO_2_/N_2_ selectivity was as high as 60, but the CO_2_ permeance was only 73 GPU, highlighting the significant challenges in achieving a defect-free, uniform coating of the selective layer on the porous hollow fiber membrane support.

Similarly, interfacial polymerization between polyoxypropylene (PEA) and TMC was used to form a TFC membrane incorporating porous graphene (pG) onto a porous flat PSf support [138]. SDS was also utilized as the surfactant. The pG layers demonstrated good adhesion with polymers and promoted hydrogen bonding, enabling the formation of an efficient molecular sieving passageway in the separation layer of the membranes. The introduction of pG increased the intermolecular periodic chain-to-chain distance of PA, resulting in looser chain packing and increased free volume. This resulted in a very high CO_2_/N_2_ selectivity of 130, but the CO_2_ permeance was only 70 GPU, despite the low thickness of the selective layer (100–200 nm). The low permeance was not clearly explained, but it might be due to the infiltration of the selective layer into the pores of the porous support.

In another study, TFC membranes composed of thin PA layers embedded with GO were prepared through a similar interfacial polymerization process [139]. The introduction of GO sheets increased the tortuosity of the polymer matrix and allowed the formation of rapid diffusion passages. The GO sheets created a permeation path perpendicular to the diffusion path, forming a maze-like structure that forced larger molecules to travel longer distances, thereby increasing overall resistance. Similar to many other GO-based membranes, the CO_2_/N_2_ selectivity was high at 41, but the CO_2_ permeance was low at 92.4 GPU.

Another functionality of GO is to mitigate the physical aging of superglassy polymers such as PTMSP over time, similar to the roles of PAFs, COFs, and MOFs. In one study [140], the PTMSP layer was modified by embedding either few-layer graphene or monolayer GO aligned parallel to the film surface. The addition of GO increased permeability, while the addition of graphene reduced it. The aging rate was found to be inversely related to film thickness, with the decay of gas permeability occurring faster for gases with higher critical temperatures. Notably, the addition of GO reduced the aging rate by 40%, attributed to the graphene fillers preventing the rearrangement of PTMSP polymer chains.

In another study [141], a simple dip-coating technique was used to fabricate TFC-MMMs comprising Pebax/GO on PVDF support. The GO sheets were aligned within a thin selective layer during the dip-coating process. Incorporating GO filler enhanced CO_2_ permeance to 413.3 GPU and CO_2_/N_2_ selectivity to 43.2. The withdrawal speed and liquid film thickness are crucial factors in the dip-coating process. The meniscus alignment with the GO laminates occurs primarily due to the high deposition force from the receding meniscus under gravity. It was noted that selecting an appropriate withdrawal speed is paramount for maximizing both selectivity and permeance.

In brief, TFC-MMMs incorporating GOs exhibited inferior separation performance, particularly in terms of CO_2_ permeance, possibly attributed to the inherently less permeable nature of GOs when compared with MOF-containing TFC-MMMs. Certain TFC-MMMs containing GOs were fabricated without utilizing a gutter layer, yet their CO_2_ permeance remained significantly lower compared with membranes with a gutter layer. The selective transport properties of GO membranes arise from their nanoscale pores and functional groups, which can selectively allow certain gas molecules to pass through while blocking others based on size, shape, and interaction with the functional groups.

## 4. IL-Containing TFC Membranes

**Figure 7:**
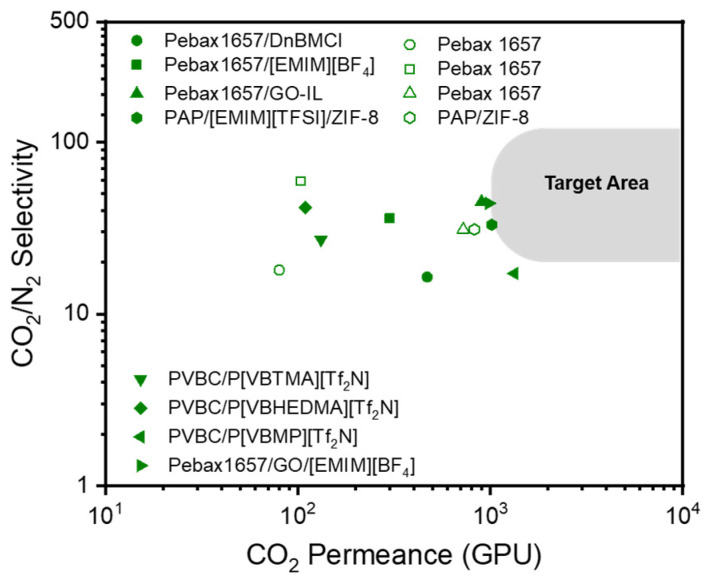
CO_2_/N_2_ selectivity vs. CO_2_ permeance of dry TFC-MMMs with various ILs reported in the literature. Unfilled symbols represent TFC membranes without the addition of ILs.

**Figure 8:**
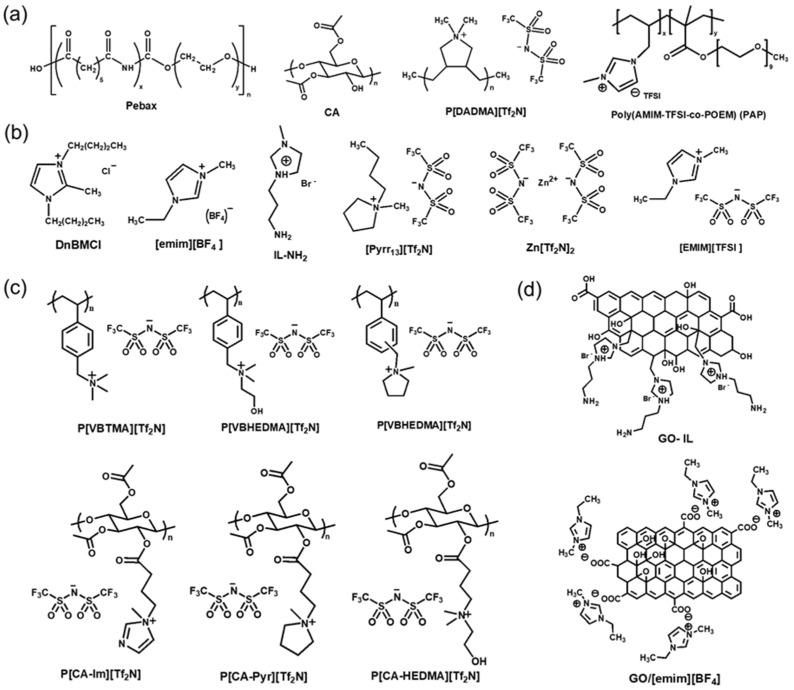
(**a**) Chemical structures of (**a**) polymer, (**b**) IL, (**c**) PIL, and (**d**) GO-IL used in the selective layer shown in Table 3 and Figure 7.

ILs, which are organic molten salts that exist in a liquid state at room temperature and under pressure, have been utilized as additives to improve the separation properties of polymer membranes [143,144,145,146,147,148,149,150,151,152,153]. ILs can capture and adsorb CO_2_ gas by leveraging the free volume between cations and anions in the lattice, thus interacting with various gases by changing the ion type [154,155]. Despite these advantages, the low capillary force holding the ILs in a composite membrane cannot withstand a high-pressure drop; therefore, they cannot meet the requirements for practical utilization. To overcome this limitation, alternative techniques, such as mixing ILs with compatible polymers, have been developed [156]. A poly(ionic liquid) (PIL) is also an effective alternative because it has both the advantages of ILs and the improved mechanical stability of polymers. The polymer backbone and IL ion type directly affect gas separation performance. The CO_2_/N_2_ separation performance of dry TFC-MMMs with various ILs reported in the literature are shown in Figure 7 and Table 3. The chemical structures of polymers and ILs are shown in Figure 8. The performance of only a few membranes containing ILs meets the commercial target for CO_2_/N_2_ separation.

In a study [157], 1,3-di-n-butyl-2-methylimidazolium chloride (DnBMCl), a rarely utilized IL, was introduced into Pebax 1657 and coated onto microporous polycarbonate (PC), which is also an uncommonly used support. The IL improved the compatibility between the selective layer and the porous support. The separation performance was moderate, achieving a CO_2_ permeance of 470 GPU and CO_2_/N_2_ selectivity of 16.4. Rather than focusing on maximizing performance, the authors highlighted the linear relationship between correction factors (β) for fractional free volumes of the TFC membranes and correction factors (α) for porosity and tortuosity of the substrate through modeling.

In another study [158], a TFC membrane was prepared using Pebax1657 blended with [emim][BF_4_] IL. A very high loading of IL at 80 wt% enabled a CO_2_ permeance of 300 GPU, a 300% increase compared with neat Pebax, and a CO_2_/N_2_ selectivity of 36. The IL’s primary function was enhancing interaction with Pebax, especially its PEO chains, through strong hydrogen bonding. This interaction reduced polymer crystallinity and increased permeance. However, the Pebax 1657/[emim][BF_4_] TFC membrane showed a 40% decrease in gas permeance after 7 months, likely due to the physical aging of the PTMSP gutter layer. Therefore, understanding the aging mechanism and developing prevention methods necessitates further study.

In a study [159], researchers used the same polymer matrix Pebax1657 but incorporated a different type of filler: GO modified with IL. This approach effectively addressed common challenges in TFC-MMM technology, such as uniform filler dispersion and the formation of defect-free membranes. The reaction between 1-(3-aminopropyl)-3-methylimidazolium bromide IL (IL-NH_2_) and GO sheets enhanced CO_2_ solubility and gas selectivity. Additionally, hydrogen bonding between the IL and amide groups in Pebax1657 promoted uniform dispersion of GO-IL. The resulting TFC membrane demonstrated good performance with a CO_2_ permeance of 900 GPU and a CO_2_/N_2_ selectivity of 45. The IL filler did not strongly interact with the PEO segments of Pebax1657 but enhanced diffusivity selectivity by providing longer and more tortuous paths for larger molecules. Overall, the introduction of IL-NH_2_ improved the compatibility at the interface between GO nanosheets and the polymer matrix.

Polyvinylbenzyl chloride (PVBC)-based PIL membranes have been fabricated as TFC to remove CO_2_ from synthetic flue gas [160]. Membranes were prepared with three cationic pendants modified from PVBC by introducing bis(trifluoromethylsulfonyl)imide ([Tf_2_N]) anions by metathesis. The first type, poly(vinylbenzyltrimethylammonium) (P[VBTMA][Tf_2_N]), was prepared by the polymerization of an IL monomer. The second type was poly(vinylbenzyl(2-hydroxyethyl)dimethylammonium)[Tf_2_N] (P[VBHEDMA][Tf_2_N])). The third type was poly(vinylbenzylmethylpyrrolidinium)[Tf_2_N] (P[VBMP] [Tf_2_N]). P[VBHEDMA][Tf_2_N], the most polar cationic pendant, exhibited the highest solubility selectivity for CO_2_. It had the highest CO_2_/N_2_ selectivity of 41 but the lowest CO_2_ permeance of 109 GPU among the three types, which can be attributed to the highest affinity of CO_2_ toward the P[VBHEDMA] cation.

Neat cellulose acetate (CA) membranes exhibit low selectivity and a risk of plasticization. CA-based membrane incorporated with IL-like pendants on the CA backbone was prepared to enhance CA separation performance [161]. CA-based membranes were prepared by the covalent grafting of cationic pendants, 1-methylimidazle (Im), N-methylpyrrolidine (Pyr), and HEDMA with anion metathesis. The use of cationic pendants with high CO_2_ affinity or hydrogen bond formation ability improved the CO_2_/N_2_ selectivity. In bifunctional materials, including Pyr and HEDMA, the HEDMA content governs the separation performance, indicating that the hydroxy-substituted cationic pendants change the interaction between the membrane network and CO_2_.

A hollow fiber TFC membrane was fabricated by blending IL [emim][BF_4_] with Pebax1657 block copolymers and adding GO [162]. The introduction of GO (up to 0.5 wt%) improved gas permeance and CO_2_/N_2_ selectivity. However, adding more GO thickened the selective layer of the TFC, and potential GO agglomeration reduced gas permeance. GO present at the interface facilitated the migration of the IL through π–π and/or cation–π interactions between GO and the IL. This IL migration to the surface, along with GO stack dispersion, was more pronounced in an alkaline environment. Consequently, a more polar film surface exhibited stronger interactions with CO_2_ molecules, which possess more than quadruple moments compared with non-polar N_2_ molecules. Under conditions of high IL content, the degree of GO sheet dispersion decreased, a process accelerated in a basic environment, and the interlayer distance between GO stacks was further reduced, diminishing the molecular sieving potential of the GO stacks. The TFC with 0.5 wt% GO and 80 wt% IL loadings demonstrated high separation performance, achieving a CO_2_ permeance of 1000 GPU and a CO_2_/N_2_ selectivity of 44.

The PIL matrix can be modified by adding an IL, and inorganic salts can be employed to enhance its suitability as a material for membrane production [163]. N-Butyl-N-methyl pyrrolidinium (Pyrr_14_) Tf_2_N was chosen as the IL, and Zn[Tf_2_N]_2_ was chosen as the salt. An increase in the Zn^2+^ content increased the CO_2_ sorption capacity; however, there was not much difference in the separation efficiency, indicating that cations participate in the effective transport of CO_2_. The maximum content of Zn^2+^ led to a 50% increase in the equilibrium solubility.

In a study [165], a modified CA grafted with PIL was proposed as a selective membrane material and coated onto a porous polyimide support. Through alkylation with butyl chloride, hydroxyl groups were substituted with pyrrolidinium cations, followed by anion exchange with bis(trifluoromethylsulfonyl)imide (Tf_2_N). The resulting membrane offers several advantages, including minimized leaching compared with ILs due to covalent bonding between ionic groups and the polymer matrix. The presence of ionic groups disrupted CA packing, while a lowered glass transition temperature indicated increased polymer chain flexibility. Despite the promising material design and synthesis, the separation performance was relatively low, particularly in permeance (below 300 GPU), attributed to the high thickness of the selective layer (1.5–5 μm).

In summary, currently, only a few TFC membranes based on ILs meet the commercial criteria for CO_2_/N_2_ separation. The lower separation performance of IL-based membranes compared with those using MOF or GO can be attributed to the liquid-like characteristics of ILs, resulting in lower gas permeance despite their enhanced CO_2_ solubility within the membrane. Among various types, ILs containing Tf_2_N anions are the most commonly used due to their strong affinity and solubility for CO_2_. The incorporation of ILs significantly improves interfacial properties and facilitates uniform coating on the porous support due to their liquid-like nature. However, many TFC membranes containing ILs still exhibit relatively thick selective layers, which results in lower gas permeance. Both PTMSP and PDMS were used, but PDMS, while offering long-term stability, has lower gas permeance. Applying a hydrophilic selective layer directly to PDMS presents challenges, leading to the widespread use of surface modification techniques, such as oxygen plasma treatment, to address these issues [166,167,168].

**Figure 9:**
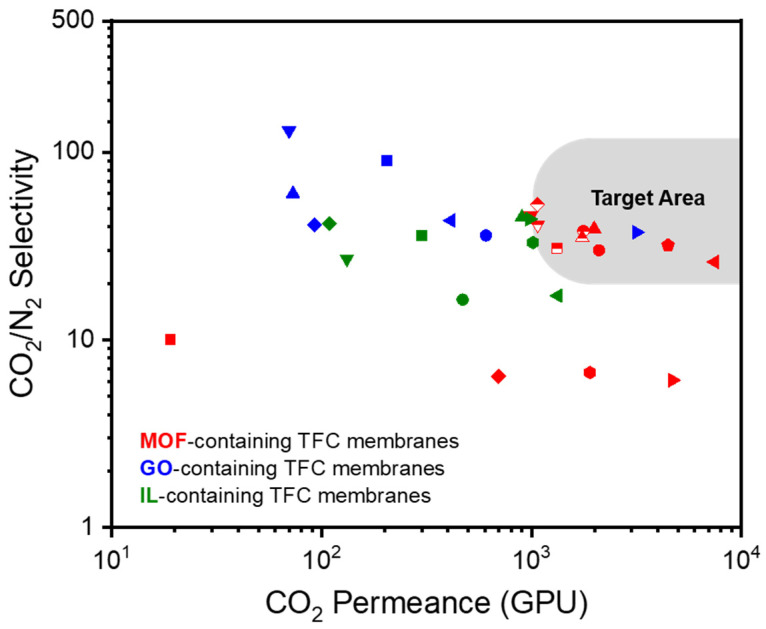
CO_2_ permeance (in GPU) vs. CO_2_/N_2_ selectivity of dry TFC membranes with three types of additives reported in the literature (red: MOF fillers, blue: GO fillers, green: IL fillers).

**Figure 10:**
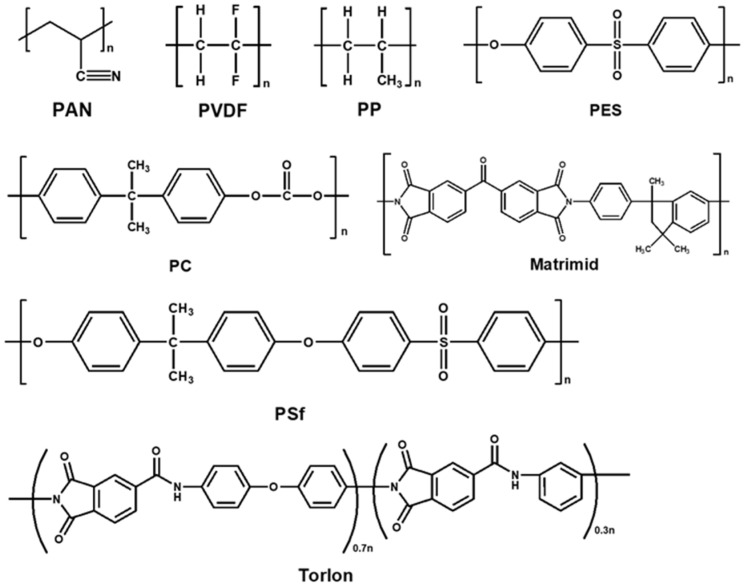
Chemical structures of polymers used as the porous support for TFC membranes with three types of additives for CO_2_/N_2_ separation.

**Figure 11:**
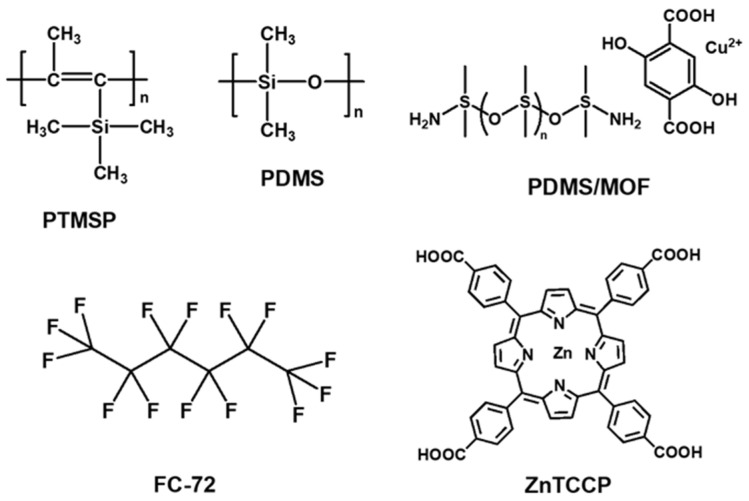
Chemical structures of materials used as the gutter layer for TFC membranes with three types of additives for CO_2_/N_2_ separation.

Figure 9 compares the gas separation performance of three types of dry TFC membranes containing additives, clearly indicating that MOF-based membranes hold the most promise for CO_2_/N_2_ separation. Overall, GO-based TFC membranes demonstrate higher CO_2_/N_2_ selectivity, while MOF-based membranes exhibit higher CO_2_ permeance in GPU. Figure 10 presents the chemical structures of the polymers used as porous support layers, while Figure 11 displays the materials utilized for the gutter layers in TFC membranes for CO_2_/N_2_ separation. Mechanically strong engineering plastics, such as PSf and PAN, are commonly used for porous supports, while high-permeability, high molecular weight polymers such as PTMSP and PDMS are widely used for gutter layers.

## 5. Conclusions and Future Perspective

As an energy-efficient CCS technology, high-permeance (in GPU) TFC membranes with various additives have been developed to overcome the limitations of pristine dense polymer membranes. The literature highlights three primary additives—MOF, GO, and IL—that have been widely used and shown to enhance gas separation performance in both permeance and selectivity. Ongoing research aims to establish economical and efficient TFC-MMM as a promising candidate for gas separation technology. Efforts continue to explore how these fillers improve material functionality and separation performance.

Several conclusions can be drawn based on this review:

(1) The majority of TFC-MMMs documented in the literature utilize flat sheets instead of hollow fibers, primarily because producing defect-free submicron-thick selective layers within hollow fibers presents challenges. Some studies have explored the fabrication of TFC hollow fiber membranes using external surface coatings instead of internal surface coatings, but the latter can pose difficulties in the development of large-scale hollow fiber membrane modules.
(2) High-performance TFC-MMMs meeting the commercial criteria for CO_2_/N_2_ separation from flue gas were mainly developed using PTMSP or PDMS gutter layers, with a minority incorporating MOFs or GOs gutter layers. This highlights the crucial role of the gutter layer in preventing excessive infiltration of the selective layer into the pores of the support. PTMSP has high permeance but undergoes physical aging over time. In contrast, PDMS has lower permeance but offers long-term stability. Being more hydrophobic than PTMSP, PDMS presents challenges when trying to apply a hydrophilic selective layer directly. As a result, surface modification of PDMS is often necessary, such as oxygen plasma.
(3) Membranes containing porous fillers like MOFs and GOs aimed to reduce non-selective interfacial defects between the fillers and polymer matrix, prevent pore blockage of the fillers by the polymer, and minimize the decrease in polymer chain mobility caused by the fillers. In contrast, membranes with ILs exhibit better interfacial contact, no pore blockage, and enhanced chain mobility due to the plasticizing effects of the ILs.
(4) In general, TFC-MMMs containing MOFs showed the best performance, followed by the membranes with GOs. The membranes with ILs showed relatively low separation performance among the three types of additives. This indicates the use of porous fillers is more effective in improving the separation performance. Additionally, careful matching between the additive and the polymer matrix is more important than the intrinsic properties of the additives.
(5) For MOF and GOs, the improved performances primarily stem from heightened diffusivity through the pores. Overall, MOF-based membranes exhibited greater CO_2_ permeance (in GPU), while GO-based membranes displayed higher CO_2_/N_2_ selectivity. Conversely, for ILs, the enhanced performance arises from increased solubility facilitated by specific interactions between ILs and CO_2_.
(6) PEO-based membranes such as Pebax are widely recognized for their extensive usage, demonstrating promising performance and suitability for commercial applications. These membranes provide several advantages: (1) ether oxygen enhances CO_2_ solubility via Lewis acid-base interactions, (2) their high solubility in mild solvents like alcohol and water allows for easy coating on porous supports without causing damage, (3) their flexible rubbery properties promote intimate contact with rigid fillers, and (4) their excellent mechanical strength facilitates easier membrane preparation using roll-to-roll processes.
(7) There are only a few studies on TFC membranes incorporating PAF or COF porous fillers as alternatives to MOFs for CO_2_ separation. Although the reported separation performance of TFC membranes containing these materials still falls short of that achieved by MOF-based MMMs, there is significant potential for growth in this research area.
(8) The combination of binary mixtures such as MOF/IL, GO/IL, and MOF/GO has often demonstrated a synergistic effect in enhancing separation performance. However, as of yet, there has been no exploration of ternary mixtures (MOF/GO/IL) in both bulk and TFC membranes for gas separation. The porous structures of MOF and GO significantly enhance gas diffusivity and size-sieving mechanisms. Conversely, IL serves as an enhancer for CO_2_ solubility and acts as a compatibilizer, improving the interfacial properties between the rigid MOF (or GO) and the soft polymer matrix. This leads to a uniform and defect-free coating of the TFC layer, further optimizing membrane performance.
(9) Surface modification and functionalization of MOFs and GO with organic groups are commonly employed to improve their interfacial properties with a polymer matrix, enhancing gas separation performance. The interactions between fillers and the matrix are primarily driven by secondary bonds like hydrogen bonding and dipole–dipole interactions. Some fillers form strong covalent bonds with the polymer matrix, resulting in more intimate contact at the interface. A major challenge, though, is the tendency of MOF and GO particles to aggregate, particularly at smaller particle sizes and higher loadings (e.g., >30%). This issue contrasts with the behavior of ILs, which do not exhibit such a tendency.
(10) The controlling factors for additives include dispersion, loading, pore size and structure, particle size, CO₂ adsorption capacity, and interfacial compatibility with the polymer. In general, the cost of MOFs, GO, and ILs is higher than that of polymer matrices due to their complex synthesis, expensive raw materials, and limited scalability. For TFC membranes containing these additives to be commercially viable, they must be cost-competitive with other CO₂ separation technologies, such as amine absorption or cryogenic distillation, to achieve large-scale adoption.

Recent advancements in TFC membranes with various additives have demonstrated their superiority in achieving significantly higher gas permeance (measured in GPU) compared with traditional micron-thick dense membranes. These TFC membranes have become a highly promising technology for CO₂ capture from flue gas. However, challenges such as scaling up the production of additives at low cost and ensuring the formation of defect-free thin layers over large areas must be addressed for successful industrial implementation. With CO₂ permeance exceeding 3000 GPU and CO₂/N₂ selectivity greater than 40, TFC membranes are poised to replace conventional membranes in industrial absorption and adsorption processes.

## Figures and Tables

**Table 1:** CO_2_/N_2_ separation performance of dry TFC-MMMs with various MOF fillers reported in the literature.

Support Material	Selective Polymer	Gutter Layer	MOF	Membrane Type	Measurement Conditions	Feed	CO_2_ Permeance (GPU)	CO_2_/N_2_ Selectivity	Ref
Torlon fiber	TFE-PPZ	N/A	SIFSIX-Cu-2i	Hollow fiber	1.3 bar, 40 °C	Flue gas	19	10	[109]
PAN	Polyactive	ZnTCPP	ZnTCPP	Flat sheet	1–5 bar, 35 °C	Pure gas	2100	32	[110]
PAN	PEG	PDMS/amorphous MOF nanosheet	N/A	Flat sheet	1 bar, 35 °C	Pure gas	1990	39	[111]
PSf	PVI-POEM copolymer	PTMSP	GO-ZIF	Flat sheet	1 bar, 25 °C	Pure gas	995.1	45.9	[53]
PAN	PIM-1	N/A	HKUST-1	Flat sheet	1 bar, 22 °C	Pure gas	696	6.4	[112]
PAN	PIM-1	PDMS/MOF	UiO-66-NH_2_	Flat sheet	1 bar, 35 °C	Pure gas	4660–7460	26–33	[113]
PAN	PTMSP/PEI	PTMSP	PAF-11	Flat sheet	2 bar, 25 °C	Pure gas	4715	6.1	[114]
PAN	PTMSP	PTMSP	PAF-11	Flat sheet	2 bar, 25 °C	Pure gas	1900	6.7	[115]
PSf	PVI-POEM copolymer	PTMSP	ZIF-8	Flat sheet	1 bar, 30 °C	Pure gas	4474	32	[116]
PSf	PGMA-co-POEM	PTMSP	UiO-66-NH_2_	Flat sheet	1 bar, 25 °C	Pure gas	1320	30.8	[77]
PSf	PGMA-co-POEM	PTMSP	MIL-140C	Flat sheet	1 bar, 25 °C	Pure gas	1768	38	[117]
			UiO-67				1745	35	
PSf	PTHFMA-co-POEM	PTMSP	UTSA-16	Flat sheet	1 bar, 30 °C	Pure gas	1070	41	[118]
PSf	PBE copolymer	PTMSP	MOF-808	Flat sheet	1 bar, 30 °C	Pure gas	1069	52.7	[119]

TFE-PPZ: poly(bis-trifluoroethoxyphosphazene), ZIF-8: zeolitic imidazole framework-8, PSf: polysulfone, PAN: polyacrylonitrile, TCPP: tetrakis(4-carboxy-phenyl)porphyrin), PEG: poly(ethylene glycol), PDMS: polydimethylsiloxane, PEI: polyethyleneimine, bim: benzimidazole, PIM: polymer of intrinsic microporosity, PVI: poly(vinyl imidazole), POEM: poly(oxyethylene methacrylate), PTMSP: poly(trimethyl silyl propyne), PAF: porous aromatic framework, PGMA: poly(glycidyl methacrylate), PTHFMA: poly(tetrahydrofurfuryl methacrylate), PBE: poly(2-[3-(2H-benzotriazole-2-yl)-4-hydroxyphenyl] ethyl methacrylate)-co-poly(oxyethylene methacrylate).

**Table 2:** CO_2_/N_2_ separation performance of dry TFC-MMMs with various GOs reported in the literature.

Support Material	Selective Polymer	Gutter Layer	GO	Membrane Type	Measurement Conditions	Feed	CO_2_ Permeance (GPU)	CO_2_/N_2_ Selectivity	Ref
PVDF	SHPAA/PVA	FC-72	pGO	Flat sheet	1.7–2 bar, 35 °C	Mixed gas (10/90 *v*/*v* of CO_2_/N_2_)	607	36	[136]
			GO-PEG				205	90	
PES	PEI-TMC-SDS	N/A	mGO	Hollow fiber	0.25 bar, 25 °C	Pure gas	73	60	[137]
PSf	PEA-TMC	N/A	pG	Flat sheet	1 bar, 30 °C	Pure gas	70	130	[138]
PSf	PA	N/A	GO	Flat sheet	2 bar, 25 °C	Pure gas	92.4	41	[139]
PP	PTMSP	N/A	GO	Flat sheet	1.3 bar, 30 °C	Pure gas	N/A	N/A	[140]
PVDF	Pebax	PTMSP	GO	Hollow fiber	2 bar, 25 °C	Pure gas	413.3	43.2	[141]
PSf	P(POEM)	PTMSP	GO-GMA	Flat sheet	1 bar, 25 °C	Pure gas	3169	37.4	[142]

PVDF: polyvinylidene fluoride, SHPAA: sterically hindered polyallylamine, PVA: polyvinyl alcohol, pGO: porous graphene oxide, PEG: poly(ethylene glycol), PES: polytehersulfone, PEI: polyethyleneimine, TMC: trimesoyl chloride, SDS: sodium dodecyl sulfate, mGO: modified GO, PEA: polyoxypropylene, PA: polyamide, PP: polypropylene, GMA: glycidyl methacrylate.

**Table 3:** CO_2_/N_2_ separation performance of dry TFC-MMMs with various ILs reported in the literature.

Support Material	Selective Polymer	Gutter Layer	IL	Membrane Type	Measurement Conditions	Feed	CO_2_ Permeance (GPU)	CO_2_/N_2_ Selectivity	Ref
PC	Pebax 1657	N/A	DnBMCl	Flat sheet	4 bar, 20 °C	Pure gas	470	16.4	[157]
PVDF	Pebax 1657	PTMSP	[emim][BF_4_]	Hollow fiber	3 bar, 35 °C	Pure gas	300	36	[158]
PVDF	Pebax 1657	PTMSP	GO-IL	Flat sheet	4 bar, 25 °C	Pure gas	900	45	[159]
Matrimid	PVBC	PDMS protective layer	P[VBTMA] [Tf_2_N]	Flat sheet	5 bar, 26 °C	Mixed gas (15/85 *v*/*v* of CO_2_/N_2_)	132	27.0	[160]
			P[VBHEDMA] [Tf_2_N]				109	41.6	
			P[VBMP] [Tf_2_N]				1334	17.2	
Matrimid	CA	PDMS protective layer	[Im][Tf_2_N]	Flat sheet	5 bar, 26 °C	Mixed gas (15/85 *v*/*v* of CO_2_/N_2_)	N/A	N/A	[161]
			[Pyr][Tf_2_N]						
			[HEDMA][Tf_2_N]						
PVDF	Pebax 1657/GO	PTMSP	[emim][BF_4_]	Hollow fiber	3 bar, 35 °C	Mixed gas (20/80 *v*/*v* of CO_2_/N_2_)	981	44	[162]
Matrimid	P[DADMA] [Tf_2_N]	PDMS protective layer	[Pyrr_14_] [Tf_2_N]	Flat sheet	1.2 bar, 26 °C	Mixed gas (15/85 *v*/*v* of CO_2_/N_2_)	N/A	N/A	[163]
			Zn[Tf_2_N]_2_						
PSf	PAP copolymer	PTMSP	[EMIM][TFSI], ZIF-8	Flat sheet	1 bar, 25 °C	Pure gas	1017	33	[164]

PC: polycarbonate, DnBMCl: 1,3-di-n-butyl-2-methylimidazolium chloride, [emim]: 1-butyl-3-methylimidazolium, mim: methylimidazolium, [Tf2N]: bis(trifluoromethylsulfonyl)imide, VBC: poly(vinylbenzyl chloride), [VBTMA]:(vinylbenzyltrimethylammonium), [VBHEDMA]: (vinylbenzyl(2-hydroxyethyl)dimethylammonium), ([VBMP]: (vinylbenzylmethylpyrrolidinium), CA: cellulose acetate, Im: 1-methylimidazle, Pyr: N-methylpyrrolidine, [HEDMA]: (2-hydroxyethyl)dimethylammonium, DADMA: diallyldimethylammonium, Pyrr: N-butyl-N-methylpyrrolidinium, PAP: Poly(AMIM-TFSI-co-POEM).

## Data Availability

Data are contained within the article.
